# Patient experiences of benzodiazepine tapering: a qualitative study of treatment in addiction care

**DOI:** 10.1186/s12888-026-08745-z

**Published:** 2026-10-08

**Authors:** Sofia Burmester, Cecilia Krüger, Johan Franck, Jeanette Westman

**Affiliations:** 1 Department of Neurobiology, Care Sciences and Society, Karolinska Institutet, Alfred Nobels allé 23, Huddinge, 141 52 Sweden; 2 The Stockholm Centre for Dependency Disorders, Stockholm Health Care Services, Region Stockholm, Friskvårdsvägen 4, Stockholm, 112 81 Sweden; 3 Department of Clinical Neuroscience, Karolinska Institutet, Tomtebodavägen 18A, Stockholm 171 77 Sweden; 4 Academic Primary Care Centre, Region Stockholm, Solnavägen 1E, Stockholm, 113 65 Sweden; 5 Department of Health Care Sciences, Marie Cederschiöld University, Folkungagatan 127, Stockholm, 116 30 Sweden

**Keywords:** Benzodiazepines, Addiction, Treatment, Substance-related disorders, Patient experiences, Qualitative research, Health care personnel, Tapering

## Abstract

**Background:**

Tapering by gradual dose reduction is a key component of treatment for individuals with addiction to benzodiazepines. Although many patients recover, treatment dropouts and relapses are common. Few studies have explored how benzodiazepine tapering is experienced from the patient perspective.

**Methods:**

This qualitative study involved semi-structured interviews with 19 adults with addiction to benzodiazepines who were undergoing tapering treatment at a publicly funded outpatient addiction clinic. Reflexive thematic analysis was used to analyze the data.

**Results:**

Participants described benzodiazepine tapering in addiction care as a process marked by substantial variations in experiences and perceived difficulty. Three themes captured central aspects of how tapering was experienced: (1) *Navigating motivation and involvement*, (2) *Replacing benzodiazepines with new coping strategies*, and (3) *Balancing support throughout treatment*.

**Conclusions:**

This study provides an experiential perspective on benzodiazepine tapering in addiction care. The findings illustrate how motivation, coping strategies, and support are closely related to participants’ experiences of discontinuation and treatment, highlighting the importance of tapering practices and interpersonal interactions for understanding engagement with treatment.

## Background

Benzodiazepines and benzodiazepine-like hypnotics (z-drugs) are often prescribed to alleviate anxiety and insomnia. Although they are different classes of medications, these drugs have comparable therapeutic effects, risks, and treatment recommendations [1] and are hereafter collectively referred to as benzodiazepines. The risk of adverse outcomes associated with benzodiazepine use has led to clinical guidelines recommending short-term use and the prioritization of other treatments [2–4]. Among the most severe potential outcomes is anxiolytic, sedative, and hypnotic use disorder [5], here referred to as addiction. Despite restrictive guidelines, benzodiazepines are sometimes prescribed for longer periods [6, 7], which increases the risk of addiction [8–10]. It is estimated that about 2% of individuals who use benzodiazepines develop addiction [11, 12], with higher rates among those with psychiatric comorbidities [13]. Benzodiazepine addiction is associated with a substantial burden on individuals, encompassing both the clinically significant impairment required for diagnosis [5], and a range of negative impacts on perceived quality of life [13].

The primary treatment approach for benzodiazepine addiction is tapering by gradual dose reduction and addressing underlying psychiatric disorders [14–16]. However, many patients drop out of treatment or experience relapse [17]. Dissatisfaction with treatment has been identified as one reason for dropout [18], underscoring the need for health care personnel to deliver care that aligns with patients’ needs and expectations. Additionally, health care personnel report significant challenges in tapering benzodiazepines, and emphasize the importance of developing effective discontinuation strategies and providing suitable alternatives to benzodiazepines for patient success [19].

Previous observational and experimental studies have investigated factors associated with treatment completion and abstinence following tapering in patients with benzodiazepine addiction [20–25]. However, many of the factors examined, including benzodiazepine dose, duration of use, age, sex, and psychiatric history, are not modifiable at the time the patient enters treatment. While these studies offer insight into individual characteristics that may influence outcomes, they provide limited information on how treatment is experienced from a patient perspective.

Qualitative methods offer a valuable means of exploring how patients experience addiction treatment, providing rich, contextual insights into how care practices are experienced and perceived. Although previous qualitative studies have examined barriers to and facilitators of discontinuing benzodiazepine use [26–36], few have focused specifically on the treatment experiences of individuals diagnosed with benzodiazepine addiction [37, 38].

Existing qualitative research has nonetheless identified several factors that influence discontinuation of benzodiazepines. Withdrawal symptoms are consistently reported as a major barrier [26–34, 36–38] while individualized tapering plans [28, 36], access to alternative symptom management strategies [26–28, 30–35], and patient support systems [26–30, 32, 33, 36] are seen as facilitators. Internal factors like motivation to quit [29, 30] and confidence in one’s ability to do so [26, 28] also appear to contribute to successful outcomes. However, these findings have not been sufficiently contextualized within the lived experiences of patients with a formal addiction diagnosis, which this study seeks to address.

Individuals diagnosed with benzodiazepine addiction may face more complex challenges than those described in studies involving individuals using benzodiazepines without a clinical diagnosis of addiction. Notably, individuals who meet fewer diagnostic criteria are more likely to successfully discontinue use compared to those who meet more criteria [25]. While the mechanism underlying this association is not yet well understood, one possibility is that individuals with a milder presentation of addiction may perceive fewer negative consequences during tapering, potentially influencing their motivation or engagement with treatment. This highlights the need to better understand the specific obstacles encountered by individuals with addiction during treatment. Moreover, current knowledge about how patients perceive support from health care personnel during treatment remains limited. Investigating the lived experiences of individuals with addiction to benzodiazepines during tapering may inform and improve treatment practices. The aim of this study was to explore how individuals with addiction to benzodiazepines experienced tapering in addiction care.

## Methods

### Study design and data collection

This interview study was conducted within a larger qualitative research project on benzodiazepine use and addiction. The present study is a primary analysis of responses to the second part of the topic guide, which addressed treatment experiences. A separate analysis of this section was planned in advance. The full topic guide, including all open-ended questions and prompts, has been published in its entirety in an earlier article [39]. Table 1 presents the questions and prompts included in the section analyzed in this paper.

A total of nineteen patients receiving treatment at an outpatient addiction clinic were included. Purposive sampling was used to include participants with varied ages, sexes, and in different stages of treatment, ensuring a broad range of experiences. Adults (≥ 18 years) diagnosed with benzodiazepine addiction, defined as sedative, hypnotic, or anxiolytic use disorder according to the Diagnostic and Statistical Manual, 5th edition (DSM-5) [5] were eligible. Those undergoing treatment for opioid use disorder or lacking sufficient proficiency in Swedish were excluded.

A multi-step recruitment process was used to minimize the risk of implicit coercion. First, treating psychiatrists or nurses identified eligible patients and asked whether they would be interested in receiving information about an interview study concerning experiences of benzodiazepine tapering. Members of the research team who were not involved in their clinical care contacted patients who had expressed interest in receiving additional information about the study, emphasizing that participation was entirely voluntary, that neither agreeing nor declining would affect ongoing treatment, and that the interview would be conducted by researchers not involved in their care (CK, JW, or a research nurse). Patients who remained interested were invited to an interview visit, where written and verbal study information was provided before written informed consent was obtained. Participants also completed a short questionnaire on their benzodiazepine use and addiction treatment. Treating clinicians were not informed which patients agreed to participate and which declined. Interviews followed a semi-structured topic guide, lasted approximately one hour (M = 57.0 min, SD = 20.4), were audio-recorded and transcribed verbatim, and were conducted between 2021 and 2023.

Transcripts were reviewed during data collection to evaluate their relevance and depth in relation to the broader research objectives of the project. In line with the concept of information power [40], the sample size was guided by the study aim, the specificity of the sample, analytic approach, and the depth of the interview material. Interviews continued until the data was deemed sufficiently rich to yield meaningful insights. The Standards for Reporting Qualitative Research [41] were followed in manuscript preparation. The study was approved by the Swedish Ethical Review Authority (Dnr. 2019–05302).

**Table 1:** Questions and prompts in the topic guide on experiences of treatment at the clinic

Questions and prompts
**Tell me about your first contact with this clinic**.
How did you experience the first contact? What was the goal of the treatment? What expectations and/or fears did you have regarding treatment?
**Tell me about your experiences of treatment at this clinic.**
Tell me about the treatment you have received/are undergoing. Can you describe your last visit at the clinic? Can you tell me about any other treatments you have received, besides tapering? What has been helpful/positive with your treatment (tapering and/or other treatment)? What has been less helpful/negative with your treatment (tapering and/or other treatment)? Is there anything that you missed in your treatment or wish was different? Have you had support outside of addiction care that has helped you during tapering treatment? Is there anything else (support, help) that you would have liked to have had during tapering treatment? In what way are you participating/involved in your treatment? How motivated have you been to participate in your treatment? Is anything different this time, compared to previous attempts to stop using benzodiazepines?
**What are your thoughts on the future?**
What could support your recovery long term? What are your thoughts on the risk for relapse? What kind of treatment/care do you think you will need in the future? What are your future plans?

### Description of treatment

Treatment at the addiction clinic followed standard care according to national and regional guidelines, and included tapering and, if necessary, psychological and/or pharmacological treatment for comorbid psychiatric disorders [14, 42]. Clinic personnel consisted of nurses, psychiatrists, and psychologists with clinical experience and/or specialization in psychiatric and addiction care. Patients were referred by other health care providers or self-referred and were assigned a primary nurse and psychiatrist. All patients underwent a clinical evaluation of somatic and psychiatric health, including laboratory and drug toxicology testing, prior to tapering. The individualized tapering protocol emphasized shared decision-making and assessment of withdrawal symptoms and well-being through weekly (or more frequent) in-person visits with their nurse, during which a regulated number of benzodiazepines were dispensed for use until the next appointment. While the standard dose reduction is 10% every one to two weeks [42], in practice, the rate is based on a plan developed collaboratively by the psychiatrist and patient at the start of treatment, and reviewed regularly. Tapering could be paused to allow for stabilization in response to substance use or worsening withdrawal or psychiatric symptoms. For example, a patient who starts off with 100 mg oxazepam daily may require a 16-week tapering schedule where they initially reduce by 10 mg per week and then taper more slowly at approximately 5 mg per week, but this should be adapted depending on individual circumstances [42]. To minimize withdrawal symptoms, patients could be switched to a long-acting formulation, and benzodiazepine doses were evenly distributed throughout the day.

### Analysis

Reflexive thematic analysis, as described by Braun and Clarke [43], was used for its suitability in identifying and interpreting patterns of meaning within qualitative data. This approach is appropriate for exploring and highlighting complex, diverse, and subjective experiences, such as those of patients undergoing addiction treatment. It supports a flexible and iterative process of theme development, emphasizing the researcher’s active and reflective engagement with the data, and acknowledging the influence of researcher subjectivity in the construction of meaning [43, 44]. Qualitative software (NVivo, version 14) was used to facilitate coding and organization.

First author (SB) read the Swedish transcripts and coded meaningful units in English. Using an inductive (i.e., data-driven) approach, SB constructed code labels that reflected both latent and semantic aspects of the text, organized the codes into initial themes and selected illustrative quotes. Coherence between quotes, codes, and themes were discussed with CK, resulting in reorganization and reconstruction of themes. All authors reviewed each revision of the manuscript, contributing perspectives based on their backgrounds. SB and JF work in addiction care, SB as a psychologist and JF as a psychiatrist. Furthermore, SB is employed at the clinic where the interviews were conducted. CK has a background in public health and anthropology, and JW in primary care and public health. In line with a biopsychosocial perspective on addiction, the authors see addiction as a multifaceted diagnosis where the interplay of biological, psychological, and social factors influence its treatment [45]. The reflexive and collaborative drafting process facilitated deeper analysis and refinement of the themes.

CK, a native English speaker, translated the quotes from Swedish to English, and SB did a back-translation. To preserve meaning and voice, quotes were modified only to remove sensitive information and add bracketed text to enhance understanding. Quotes were pseudonymized; each participant was assigned a random code.

## Results

The study population consisted of ten men and nine women with a mean age of 53.4 years (SD = 14.6), see Table 2. On average, participants were in treatment for 7.6 months (SD = 5.1), with 52.6% still tapering at the time of the interview. Alprazolam was the most used benzodiazepine at treatment initiation (*n* = 10), although more than half (57.9%) used two or more benzodiazepines/z-drugs. The most common combination was alprazolam together with either zopiclone and/or zolpidem (*n* = 6). Participants had used benzodiazepines for 2 to 45 years (M = 16.6, SD = 13.7) before treatment, and 31.6% had previously sought treatment for benzodiazepine addiction. Lifetime illicit use (i.e., obtainment outside the health care sector) was reported by more than one third (36.8%).

**Table 2:** Characteristics and treatment variables of the study population

Variable	Total sample, N=19
**Age in years**	
Mean (SD)	53.4 (14.6)
Range	25-75
**Sex**	
Men, n (%)	10 (52.6)
Women, n (%)	9 (47.4)
**Duration of use in years**	
Mean (SD)	16.6 (13.7)
Range	2-45
**Benzodiazepines and z-drugs used at treatment initiation**	
Alprazolam, n (%)	10 (52.6)
Clonazepam, n (%)	2 (10.5)
Diazepam, n (%)	4 (21.1)
Oxazepam, n (%)	5 (26.3)
Zolpidem, n (%)	5 (26.3)
Zopiclone, n (%)	8 (42.1)
**Use of ≥2 benzodiazepines/z-drugs**	
Yes, n (%)	11 (57.9)
**Lifetime illicit use** ^**1**^	
Yes, n (%)	7 (36.8)
**Months in addiction treatment at the clinic**	
Mean (SD)	7.6 (5.1)
Range	2-22
**Phase of treatment** ^**1**^	
Ongoing tapering, n (%)	10 (52.6)
Completed tapering, n (%)	9 (47.4)
**Previous treatment for benzodiazepine addiction**	
Yes, n (%)	6 (31.6)

Abbreviations: SD, standard deviation

^1^ Ascertained from the interviews

Participants’ experiences of benzodiazepine tapering revealed substantial variability in perceived difficulty, spanning from transient and manageable to enduring and severely impairing. Participants’ multifaceted experiences during tapering treatment are described in three themes: (1) *Navigating motivation and involvement*, (2) *Replacing benzodiazepines with new coping strategies*, and (3) *Balancing support throughout treatment*.

### Theme 1. Navigating motivation and involvement

Many participants entered treatment with ambivalence, torn between hope and apprehension, uncertain about the treatment process and its effectiveness. This ambivalence was reflected in their motivation, which fluctuated from treatment initiation to after discontinuation of benzodiazepines: “*Your motivation*,* that’s the most important thing*,* but it’s not so easy.*” (P16). Some participants had high motivation and were determined to succeed from the beginning, bolstered by hope for a better future and a life without benzodiazepines. This vision motivated them, reinforcing their belief in recovery, supporting their sustained commitment to treatment despite its challenges, and lowering their perceived risk of relapse.

I mean, it’s been so obvious to me, that I wanted to quit. There was no hesitation from my side, and I haven’t wavered in that in any way, and then [all that’s left] is to just do it. (P14)

For other participants, the fear of feeling unwell and returning to benzodiazepine use made dose reduction feel particularly daunting: “*You need to grit your teeth and steel yourself*,* I think. Otherwise*,* it won’t work […] it is a damned uncomfortable feeling to stop. It’s not fun at all and you need to be ready for it.”* (P05). Interactions with health care personnel motivated participants who started out as ambivalent to engage with treatment: *“They deserve credit for the fact that I can see some kind of a life where I don’t need to use benzos.”* (P10). Participants highlighted the importance of interactions characterized by empathy, respect, encouragement, and attentiveness to their progress, which fostered a sense of motivation and hope. Participants described such interactions as influencing their perseverance during challenging phases of tapering.

I became more and more motivated the further along I went, so now I’m pretty… very motivated to not start [using benzodiazepines] again, but in the beginning I wasn’t motivated at all. (P07)

Although withdrawal symptoms were generally expected and persisted even post-discontinuation of benzodiazepines, their severity significantly shaped participants’ treatment experiences and influenced their motivation to continue. Participants who experienced no, few, or temporary withdrawal symptoms generally felt that treatment was progressing well: “*I guess I felt a little bit when decreasing [the dose]*,* but it was surprisingly little.*” (P15). Some participants even wanted to expedite tapering: “*Hello*,* can we … speed this up a little*?” (P09). In contrast, participants who experienced more intense withdrawal symptoms described significant distress.

I mean, there’s a lot of side effects just from stopping […] I couldn’t sleep. I had so much anxiety that I was… I thought I was starting to lose my mind. (P07)

Participants described experiences where greater involvement in treatment decisions helped motivate them during periods of symptom-related difficulty. Collaborative processes involving flexible tapering schedules, where the pace was adjusted or dose reductions postponed in response to worsening symptoms, were appreciated by participants.

Yeah, I guess it’s been pretty flexible. It almost always been up to me, when I’m going to taper and such […] And sometimes, maybe I felt like, now it’s been a couple of difficult days, then maybe I’ll stop on that dose for three more days, or something like that. So, it’s been nice and good that it’s been a little up to me, that it hasn’t been a set schedule. (P12)

When participants perceived there was little room to question or change the tapering schedule based on their individual needs, they described the treatment structure as rigid and impersonal: “*So it feels like… almost like I’ve been held hostage.*” (P17). They also expressed frustration with communication, especially when they felt uninformed about treatment goals, plans, or what to expect. Combined with withdrawal symptoms, a perceived lack of information and flexibility amplified feelings of helplessness. This, in turn, sometimes strained communication, particularly in situations where participants felt pressured to reduce their dose.

And today they questioned, like, if I really want to quit or not. Like: “Fuck it, get rid of them today, I don’t have any problems with that, but I don’t want to feel this awful.” (P18)

### Theme 2. Replacing benzodiazepines with new coping strategies

For many participants, tapering meant letting go of something that had long served as a stabilizing force in their lives. Benzodiazepines were participants’ primary strategy for managing symptoms of mental illness, often over the course of several decades: “… *well*,* I guess it’s been more like a crutch*,* that it serves a certain function for a little while … I mean*,* it’s some sort of help*,* kind of*,* if even that.”* (P04). Letting go of this crutch meant surrendering control over their (often only) coping strategy without knowing what would replace it.

"Help, they’re taking my medication.” […] I thought: “how am I going to make it without access to benzos?" (P10)

The idea of no longer having benzodiazepines to rely on was especially challenging for participants whose underlying mental health problems persisted or worsened during treatment: “*I was very scared to let go of that last tiny [dose].”* (P11). In moments of distress, participants sometimes resorted to using higher than prescribed benzodiazepine doses during tapering or turned to alcohol. Even after discontinuation of use, participants had to actively resist the urge to resume using benzodiazepines: “*There have been a few nights where I’ve thought about it: ‘Maybe I should just take one pill anyways?’*” (P04). Relapse was not seen as a distant possibility, but as a real and immediate concern.

It’s not that I want to […] but if I get that much anxiety again and that much stress again, then I will start using again. It feels like there’s really nothing that can stop it from happening. (P07)

During treatment, participants realized that they needed to adopt alternative strategies for managing symptoms. Participants often entered treatment with expectations of support in finding new coping strategies: “*I mean*,* if you get rid of something then you maybe need to add something else*,* whether it be yoga or whatever the hell else*,* to have a bearable life.*” (P18), and appreciated being offered several different kinds of treatment options. Access to psychological treatment, whether initiated during tapering or scheduled after discontinuation of benzodiazepines, was described as a primary strategy for addressing reasons for benzodiazepine use and developing new coping strategies, and was also described as encouraging participants to remain in treatment.

So I’m in touch with a psychologist now, to, like, start addressing the underlying problems that made me start with benzos in the first place. […] so it’s [treatment] has been about more than just tapering out, and I guess it is now too, and that’s part of why I still go here [the clinic]. (P12)

However, some participants declined psychological treatment due to anxiety, time constraints, or prior negative experiences. Other participants reported delays, unclear availability, or timing issues that made psychological treatment an ineffective option:

I saw a psychologist for a bit. I’m supposed to work with my anxiety, but I was still on such a high dose then, so I was still totally dazed in the head. I couldn’t … so we didn’t get anywhere. (P07)

Fear of relapse also made participants more open to other support options, including Narcotics Anonymous or non-addictive medication: “*I mean*,* I’m going to take whatever chance I can get that makes it so I don’t end up addicted again.*” (P13). Without an effective replacement for benzodiazepines, participants described life as restricted and symptom-filled, heightening anxiety about the future:

The future makes me feel panicked or gives me anxiety. I mean, I hope that I’ll feel good, but it starts to feel meaningless in a way after so many years of … of having felt so poorly. (P08)

### Theme 3. Balancing support throughout treatment

Over the course of treatment, participants emphasized the importance of support from health care personnel. Participants valued personnel not just for their medical expertise—“*I feel like*,* you know*,* just knowing that they are super knowledgeable*,* and that*,* that makes me feel safe in some way.*” (P03)—but for their ability to provide guidance in times of uncertainty. The care and attention of personnel were experienced as enhancing participants’ confidence in their ability to quit.

Because if it had only been, like, that I had come here [the clinic] and: “Here you have this [benzodiazepine] and I’ll see you again in three days” […] If there hadn’t been this kind of evaluation… and they ask how I’m feeling and how they could make it easier to take a urine test and that kind of thing, then it would have been so much easier to just: “Yeah, well whatever. I can just take these [benzodiazepines] here and then I can go buy [more benzodiazepines]”. (P13)

Participants described forming close relationships with health care personnel, particularly their nurse, which shaped how they experienced tapering. Within these relationships, participants felt able to laugh together and speak openly about difficult topics, including disclosing missteps (e.g., using a higher dose of benzodiazepines) or expressing dissatisfaction without fear of being denied care. Interactions with personnel were sometimes described as the core component of treatment. Conversations that helped normalize their experiences alleviated anxiety and made participants feel better.

I think it’s been really nice to be able to come here and talk to someone, say how I’m feeling and be told that I’m not crazy. (P09)

When participants felt listened to and understood they became more engaged in treatment: “*I mean*,* I could hear that they understood that I was scared*,* because that’s what it is*,* fear*,* when one becomes so angry and so…*” (P15). Some participants described that personnel understood what they were going through better than their own families and demonstrated genuine care—they “*really care about you*” (P03)—by being responsive to their needs and circumstances.

I feel fully supported, like if an obstacle were to come up, I would get help to overcome it. (P01)

However, participants described a blurred line between support that felt appropriate in type and amount, and support that was perceived as unwanted. Brief clinic visits and difficulty reaching personnel between appointments left participants feeling alone, particularly during periods of intensified withdrawal symptoms. Experiences of unmet promises or impersonal support further contributed to feelings of helplessness.

For example, they say my name: “[name], we’re helping you and we’re helping you, we’re helping you”, but when you leave you’re like: “how are they going to help me?” (P16).

A perceived lack of responsiveness to their needs contributed to emotional distress and, for some participants, prompted doubts about the value of continuing treatment. In other cases, support was experienced as intrusive rather than helpful. When personnel made decisions unilaterally or pressured participants to engage in activities or therapies they did not wish to pursue, participants described feeling that their perspectives were overlooked.

And they have helped me quite a… they have helped me a little too much sometimes. It has sometimes stressed me out a little. (P09).

Expectations surrounding the intensity of support also influenced perceptions of care. Many participants appreciated long-term follow-up after discontinuation of benzodiazepines, as well as the option to contact personnel between visits: “*I feel like they always have my back. They’re a phone call away if I need anything.*” (P06). Others experienced a gradual decline in support over time, which they perceived as challenging during recovery. Relying on health care resources could also be experienced as taxing: “*I feel costly right now*,* actually. Honestly*,* it feels like I’m a burden*.” (P17). To avoid these feelings, some participants withheld their struggles from personnel, or even proposed reducing the number of clinic visits, which ultimately led to a decline in perceived support.

Participants also highlighted the importance of practical support from loved ones, for example, driving them to clinic visits, assisting with medication management, or limiting access to illicit benzodiazepines. This tangible support was particularly valued during rough periods. Loved ones’ reassurance also helped participants stay engaged with the treatment and made them feel less alone: “‘*Damn*,* you’re doing good*,* mom. […] We are so proud of you for doing this.’*” (P06). Some even expressed that, in the absence of support from loved ones, they probably could not have completed treatment or would have required additional support from health care personnel.

I would never have made it if my family hadn’t helped me, that’s for sure. […] I mean… then I would have needed to come here [the clinic] every day. Or the primary care center, or whatever. But I would never have been able to do this by myself, never. Never. (P17)

## Discussion

This study is among the first to explore how individuals with addiction to benzodiazepines perceived and experienced tapering in addiction care. The findings suggest that tapering was not merely perceived as medication reduction, but rather as a multifaceted and emotionally demanding process that required motivation, new coping strategies, and finding the right balance of support to successfully discontinue and sustain abstinence. While certain patient-related factors are fixed at the point of treatment initiation, health care delivery is not. Participants’ accounts highlight how treatment practices and interactions were closely tied to their overall experience of benzodiazepine discontinuation and level of engagement with treatment. By focusing on patient-identified aspects, this study offers valuable insights that may inform and improve treatment practices.

The first theme, *Navigating motivation and involvement*, highlights how motivation to discontinue benzodiazepines emerged as a dynamic process closely linked to participants’ ability to manage fluctuating withdrawal symptoms, their perceived control over tapering, and interactions with health care personnel. Those with minimal symptoms found tapering manageable, while others faced significant difficulties, echoing earlier studies [26, 28, 34, 37]. These findings suggest that participants with severe withdrawal symptoms or marked ambivalence to treatment need closer attention to address flagging motivation. When interactions were perceived as empathetic and respectful, and when care practices were responsive and attuned to individual circumstances and progress, participants experienced motivation and engagement in treatment. This aligns with previous research describing motivation to discontinue benzodiazepines as a dynamic and context-dependent process [29].

Involvement in decision-making and flexibility in tapering schedules were experienced as particularly important during periods of heightened difficulty, as it enhanced participants’ sense of control and sustained their commitment to treatment. Conversely, inflexible tapering plans and limited autonomy, particularly during periods of personal struggle, were described as challenging, reflecting patterns observed in earlier research [27, 36]. Although clinical data suggest that overly prolonged tapering schedules may increase psychological strain [15], this study indicated that patients require individualized tapering schedules. Together, these findings show that motivation during tapering is an iterative process that develops over time through interactions between symptoms, perceptions of care (including tapering schedules), and health care personnel, rather than being a prerequisite for treatment engagement.

The second theme *Replacing benzodiazepines with new coping strategies*, explores how benzodiazepines had long served as a means of managing symptoms related to underlying mental health problems and the need for viable alternatives. Participants described that giving up benzodiazepines was particularly difficult in the absence of alternative coping strategies, shaping their vulnerability and perceived risk of relapse. While previous research has emphasized the importance of alternative coping strategies to facilitate benzodiazepine discontinuation [23, 26–28, 30–32, 34–36], this study adds a more nuanced understanding of how engagement with new strategies were experienced as mixed and context-dependent.

The challenges that participants described were twofold. Some participants, who were open to replacement strategies, including psychological treatment, were unable to meaningfully engage due to barriers such as poor timing or inaccessibility. Others felt that the support that was offered to them was insufficiently aligned with their wants or needs, resulting in a missed opportunity for intervention. Together, these findings underscore that the experience of trying to replace benzodiazepines involves more than introducing alternatives, and that meaningful engagement depends on whether alternatives are offered at the right time and in a way that aligns with an individual’s preference.

The third theme, *Balancing support throughout treatment* emphasized the importance of feeling seen and heard, and that support was most helpful when its type, timing, and intensity were aligned with participants’ needs. Relationships and interactions with health care personnel were central to participants feeling supported during tapering, although not all forms of support were perceived as equally helpful. Consistent with previous studies [27, 28, 30, 33], insufficient support, such as having questions left unanswered, receiving limited guidance, or perceiving that health care personnel were unavailable, was experienced as distressing and contributed to feelings of isolation. In contrast, interactions characterized by knowledgeability, genuine interest, understanding, and availability facilitated discontinuation [27, 29, 30]. Importantly, this study adds a nuanced account of variability in how support was perceived, where a mismatch sometimes occurred between expected or desired support and that which they received. These results highlight that an open dialog where health care personnel are receptive to patients’ feedback and needs might improve patient experiences of support.

Another important finding concerns the role of loved ones during tapering and the balance between personal and professional support. Although previous studies have shown that involvement of loved ones may facilitate benzodiazepine discontinuation [28–30], this study further described how the presence or absence of support from loved ones affected how participants relied on health care and gave specific examples where loved ones provided complementary care. In situations where support from loved ones was limited, greater support from health care personnel was perceived as necessary.

### Strengths and limitations

The variation in participants’ treatment duration and phase (i.e., ongoing or completed tapering) contributed to a rich and diverse set of experiences. All participants’ experiences were treated as equally valuable in the analysis. However, it should be noted that because all participants were either undergoing or had completed tapering, the results may not reflect the experiences of individuals who dropped out of treatment.

The study was conducted by researchers with experience in addiction care. One member of the research group (SB) was employed as a psychologist at the clinic where participants received treatment. However, SB did not participate in the recruitment or interviewing of participants and only reviewed pseudonymized transcripts for research purposes. The recruitment and interviews were conducted by study personnel external to the clinic, and none of the researchers were involved in or had any influence over participants’ tapering of benzodiazepines. Participants received information about the study prior to providing written informed consent, including that the interviews would be conducted by study personnel outside the clinic and that participation would not affect treatment. Transcripts were pseudonymized before analysis.

Given that participants in this study were recruited from a single outpatient addiction clinic specialized in tapering, the experiences described are specific to this setting. However, tapering is a common treatment and many of their experiences are likely relevant across different addiction care settings. Although some participants described use of other substances during the interviews, particularly alcohol, the exclusion of patients with opioid use disorder and the limited data on other substance use disorders implies that experiences of polysubstance users may not be fully captured. Moreover, while interview participants commonly described mental illness as a reason for initiating benzodiazepine use [46], information on comorbid psychiatric disorders was not systematically collected.

The analysis and results were shaped by the clinical perspectives of SB and the co-authors. SB’s experience of listening to patients’ stories as a psychologist may have influenced the interpretation. However, through collaborative discussions and revisions among the authors, a nuanced presentation of participants’ experiences was achieved. Applying reflexivity and using rich data enhanced the trustworthiness and quality of the analysis. Few previous studies have included participants with a formal diagnosis of addiction, and none within a Nordic context. Thus, this study contributes new knowledge about lived experiences of patients undergoing treatment for benzodiazepine addiction and provides insights relevant to this healthcare context.

## Conclusion

This study explored how patients with benzodiazepine addiction experienced tapering in addiction care. The findings provide an experiential perspective of addiction treatment and describe how motivation, new coping strategies, and support were central aspects of participants’ tapering experiences and engagement. Furthermore, these experiences were shaped through interactions with health care personnel, treatment practices, and withdrawal and psychiatric symptoms. By focusing on patient perspectives, this study offers valuable insight into the challenges of benzodiazepine tapering, with relevance for informing and improving tapering practices.

## Data Availability

The datasets generated and analyzed during the study are not publicly available due to the sensitivity of the data but are available from the corresponding author on reasonable request subject to applicable ethical approvals.

## References

[1] Brandt J, Bressi J, Lê ML, Neal D, Cadogan C, Witt-Doerring J, Witt-Doerring M, Wright S. Prescribing and deprescribing guidance for benzodiazepine and benzodiazepine receptor agonist use in adults with depression, anxiety, and insomnia: an international scoping review. EClinicalMedicine. 2024;70:102507. 10.1016/j.eclinm.2024.102507 PMC10955669 38516102

[2] National Institute for Health and Care Excellence. Anxiety disorders: quality standard (QS53). 2014. https://www.nice.org.uk/guidance/qs53. Accessed 7 Oct 2025.

[3] National Institute for Health and Care Excellence. Guidance on the use of zaleplon, zolpidem and zopiclone for the short-term management of insomnia: technology appraisal guidance. 2004. https://www.nice.org.uk/guidance/ta77. Accessed 7 Oct 2025.

[4] National Board of Health and Welfare. National guidelines for care in depression and anxiety disorders: support for control and management [Nationella riktlinjer för vård vid depression och ångestsyndrom. Stöd för styrning och ledning]. 2021. https://www.socialstyrelsen.se. Accessed 18 Jan 2024.

[5] American Psychiatric Association. Diagnostic and statistical manual of mental disorders. Fifth ed. Washington DC: American Psychiatric Association; 2013.

[6] Kurko TA, Saastamoinen LK, Tahkapaa S, Tuulio-Henriksson A, Taiminen T, Tiihonen J, Airaksinen MS, Hietala J. Long-term use of benzodiazepines: Definitions, prevalence and usage patterns - a systematic review of register-based studies. Eur Psychiatry. 2015;30(8):1037–47. 10.1016/j.eurpsy.2015.09.003 26545257

[7] Isomura K, Wang X, Chang Z, Hellner C, Hasselstrom J, Ekheden I, Jayaram-Lindstrom N, Lichtenstein P, D’Onofrio BM, Mataix-Cols D, et al. Factors associated with long-term benzodiazepine and Z-drug use across the lifespan and 5-year temporal trajectories among incident users: a Swedish nationwide register-based study. Eur J Clin Pharmacol. 2023;79(8):1091–105. 10.1007/s00228-023-03515-2 PMC10361867 37294340

[8] Fenton MC, Keyes KM, Martins SS, Hasin DS. The role of a prescription in anxiety medication use, abuse, and dependence. Am J Psychiatry. 2010;167(10):1247–53. 10.1176/appi.ajp.2010.09081132 PMC3756550 20595413

[9] Schifano F, Chiappini S, Corkery JM, Guirguis A. An Insight into Z-Drug Abuse and Dependence: An Examination of Reports to the European Medicines Agency Database of Suspected Adverse Drug Reactions. Int J Neuropsychopharmacol. 2019;22(4):270–7. 10.1093/ijnp/pyz007 PMC6441128 30722037

[10] Peng L, Morford KL, Levander XA. Benzodiazepines and Related Sedatives. Med Clin North Am. 2022;106(1):113–29. 10.1016/j.mcna.2021.08.012 34823725

[11] McHugh RK, Votaw VR, Trapani EW, McCarthy MD. Prevalence and correlates of the misuse of z-drugs and benzodiazepines in the National Survey on Drug Use and Health. Front Psychiatry. 2023;14:1129447. 10.3389/fpsyt.2023.1129447 PMC10033696 36970272

[12] Blanco C, Han B, Jones CM, Johnson K, Compton WM. Prevalence and Correlates of Benzodiazepine Use, Misuse, and Use Disorders Among Adults in the United States. J Clin Psychiatry. 2018;79(6):18m12174. 10.4088/JCP.18m12174 PMC10309967 30403446

[13] Votaw VR, Geyer R, Rieselbach MM, McHugh RK. The epidemiology of benzodiazepine misuse: A systematic review. Drug Alcohol Depend. 2019;200:95–114. 10.1016/j.drugalcdep.2019.02.033 PMC6639084 31121495

[14] National Board of Health and Welfare. National guidelines for care and support in cases of addiction and dependence: support for control and management [Nationella riktlinjer för vård och stöd vid missbruk och beroende. Stöd för styrning och ledning]; 2019.

[15] Soyka M. Treatment of Benzodiazepine Dependence. N Engl J Med. 2017;376(12):1147–57. 10.1056/NEJMra1611832 28328330

[16] Brunner E, Chen CA, Klein T, Maust D, Mazer-Amirshahi M, Mecca M, Najera D, Ogbonna C, Rajneesh KF, Roll E, et al. Joint Clinical Practice Guideline on Benzodiazepine Tapering: Considerations When Risks Outweigh Benefits. J Gen Intern Med. 2025;40(12):2814–59. 10.1007/s11606-025-09499-2 PMC12463801 40526204

[17] Ashton H. The diagnosis and management of benzodiazepine dependence. Curr Opin Psychiatry. 2005;18(3):249–55. 10.1097/01.yco.0000165594.60434.84 16639148

[18] Vorma H, Naukkarinen H, Sarna S, Kuoppasalmi K. Treatment of out-patients with complicated benzodiazepine dependence: comparison of two approaches. Addiction. 2002;97(7):851–9. 10.1046/j.1360-0443.2002.00129.x 12133124

[19] Sirdifield C, Anthierens S, Creupelandt H, Chipchase SY, Christiaens T, Siriwardena AN. General practitioners’ experiences and perceptions of benzodiazepine prescribing: systematic review and meta-synthesis. BMC Fam Pract. 2013;14:191. 10.1186/1471-2296-14-191 PMC4028802 24330388

[20] Ashton H. Benzodiazepine withdrawal: outcome in 50 patients. Br J Addict. 1987;82(6):665–71. 10.1111/j.1360-0443.1987.tb01529.x 2886145

[21] Golombok S, Higgitt A, Fonagy P, Dodds S, Saper J, Lader M. A follow-up study of patients treated for benzodiazepine dependence. Br J Med Psychol. 1987;60:141–9. 10.1111/j.2044-8341.1987.tb02724.x 2887197

[22] Rickels K, Case WG, Schweizer E, Garcia-Espana F, Fridman R. Long-term benzodiazepine users 3 years after participation in a discontinuation program. Am J Psychiatry. 1991;148(6):757–61. 10.1176/ajp.148.6.757 2035717

[23] O’Connor K, Marchand A, Brousseau L, Aardema F, Mainguy N, Landry P, Savard P, Leveille C, Lafrance V, Boivin S, et al. Cognitive-behavioural, pharmacological and psychosocial predictors of outcome during tapered discontinuation of benzodiazepine. Clin Psychol Psychother. 2008;15(1):1–14. 10.1002/cpp.556 19115423

[24] Vorma H, Naukkarinen H, Sarna S, Kuoppasalmi K. Long-term outcome after benzodiazepine withdrawal treatment in subjects with complicated dependence. Drug Alcohol Depend. 2003;70(3):309–14. 10.1016/s0376-8716(03)00014-0 12757968

[25] Vorma H, Naukkarinen HH, Sarna SJ, Kuoppasalmi KI. Predictors of benzodiazepine discontinuation in subjects manifesting complicated dependence. Subst Use Misuse. 2005;40(4):499–510. 10.1081/ja-200052433 15830732

[26] Lynch T, Ryan C, Cadogan CA. I just thought that it was such an impossible thing’: A qualitative study of barriers and facilitators to discontinuing long-term use of benzodiazepine receptor agonists using the Theoretical Domains Framework. Health Expect. 2022;25(1):355–65. 10.1111/hex.13392 PMC8849267 34862703

[27] Oldenhof E, Mason T, Anderson-Wurf J, Staiger PK. Role of the prescriber in supporting patients to discontinue benzodiazepines: a qualitative study. Br J Gen Pract. 2021;71(708):e517–27. 10.3399/BJGP.2020.1062 PMC8216269 33950855

[28] Parr JM, Kavanagh DJ, Young RM, McCafferty K. Views of general practitioners and benzodiazepine users on benzodiazepines: a qualitative analysis. Soc Sci Med. 2006;62(5):1237–49. 10.1016/j.socscimed.2005.07.016 16143440

[29] Coteur K, Van Nuland M, Schoenmakers B, Van den Broeck K, Anthierens S. At the time I only wanted to relieve stress: Exploring motivation for behaviour change in long-term hypnotic users. Heliyon. 2023;9(5):e16215. 10.1016/j.heliyon.2023.e16215 PMC10205632 37234622

[30] Lynch T, Ryan C, Huff C, Foster DE, Cadogan C. We need more support and doctors that understand the process of tapering … A content analysis of free-text responses to a questionnaire on discontinuing long-term benzodiazepine receptor agonist use. Health Expect. 2024;27:e13962. 10.1111/hex.13962 PMC10771803 39102711

[31] Cook JM, Biyanova T, Masci C, Coyne JC. Older patient perspectives on long-term anxiolytic benzodiazepine use and discontinuation: a qualitative study. J Gen Intern Med. 2007;22(8):1094–100. 10.1007/s11606-007-0205-5 PMC2305752 17492325

[32] Fixsen AM, Ridge D. Stories of Hell and Healing: Internet Users’ Construction of Benzodiazepine Distress and Withdrawal. Qual Health Res. 2017;27(13):2030–41. 10.1177/1049732317728053 28891380

[33] Fixsen AM. I’m Not Waving, I’m Drowning: An Autoethnographical Exploration of Biographical Disruption and Reconstruction During Recovery From Prescribed Benzodiazepine Use. Qual Health Res. 2016;26(4):466–81. 10.1177/1049732315576496 25800715

[34] Takamine L, Krein SL, Ratliff E, Strominger J, Virk A, Maust DT. Examining Adult Patients’ Success with Discontinuing Long-term Benzodiazepine Use: a Qualitative Study. J Gen Intern Med. 2024;39(2):247–54. 10.1007/s11606-023-08385-z PMC10853089 37653209

[35] Barter G, Cormack M. The long-term use of benzodiazepines: patients’ views, accounts and experiences. Fam Pract. 1996;13(6):491–7. 10.1093/fampra/13.6.491 9023523

[36] Van Ngoc P, Ceuterick M, Belche JL, Scholtes B. I haven’t discussed anything with anyone’: lived experience of long-term users of benzodiazepine receptor agonists regarding their treatment for substance use disorder. Int J Qualitative Stud Health Well-Being. 2024;19:2424013. 10.1080/17482631.2024.2424013 PMC11574945 39552059

[37] Liebrenz M, Gehring MT, Buadze A, Caflisch C. High-dose benzodiazepine dependence: a qualitative study of patients’ perception on cessation and withdrawal. BMC Psychiatry. 2015;15:116. 10.1186/s12888-015-0493-y PMC4443548 25968120

[38] Kapadia N, Fox D, Rowlands G, Ashworth M. Developing primary care services for high-dose benzodiazepine-dependent patients: A consultation survey. Drugs: Educ Prev Policy. 2007;14(5):429–42.

[39] Burmester S, Krüger C, Franck J, Hedlund Lindberg M, Westman J. At the tipping point: Patient experiences of addiction to benzodiazepines and motivation to seek treatment. J Subst Use Addict Treat. 2024;167:209508. 10.1016/j.josat.2024.209508 39243978

[40] Malterud K, Siersma VD, Guassora AD. Sample Size in Qualitative Interview Studies: Guided by Information Power. Qual Health Res. 2016;26(13):1753–60. 10.1177/1049732315617444 26613970

[41] O’Brien BC, Harris IB, Beckman TJ, Reed DA, Cook DA. Standards for reporting qualitative research: a synthesis of recommendations. Acad Med. 2014;89(9):1245–51. 10.1097/ACM.0000000000000388 24979285

[42] Region Stockholm. Clinical guidelines and support for healthcare providers: prescription drug addiction [Kunskapsstöd för vårdgivare: läkemedelsberoende] 2013. https://www.kunskapsstodforvardgivare.se/omraden/psykisk-halsa/regionala-vardprogram/lakemedelsberoende. Accessed 28 April 2025.

[43] Braun V, Clarke V. Thematic Analysis: A Practical Guide. Thousand Oaks, CA: SAGE Publications Ltd; 2022.

[44] Braun V, Clarke V. One size fits all? What counts as quality practice in (reflexive) thematic analysis? Qualitative Res Psychol. 2021;18(3):328–52.

[45] Skewes MC, Gonzalez VM. The Biopsychosocial Model of Addiction. In: Miller PM, editor. Principles of Addiction: Comprehensive Addictive Behaviors and Disorders. Volume 1. San Diego: Elsevier Science and Technology; 2013. pp. 61–70.

[46] Krüger C, Hedlund Lindberg M, Burmester S, Franck J, Westman J. Experiences of long-term benzodiazepine use and addiction amid changes in guidelines for the prescription of narcotic drugs: a qualitative study. BMC Public Health. 2025;25(1):2652. 10.1186/s12889-025-23920-9 PMC12326687 40764568

